# Supplementary material for: Microbial Strategies for Survival in the Glass Sponge Vazella pourtalesii
Source: mSystems. 2020 Aug 11;5(4):e00473-20. doi: 10.1128/mSystems.00473-20 (PMC7426153; doi:10.1128/mSystems.00473-20)
Supplement: TEXT S1 [file mSystems.00473-20-s0001.doc]

**Text S1 Detailed description of metabolic features of SAR324 and Crenarchaeota.**

# Microbial strategies for survival in the glass sponge *Vazella pourtalesii*

Bayer K^1^*, Busch K^1^*, Kenchington E^2^, Beazley L^2^, Franzenburg S^3^, Michels J^4^, Hentschel U^1^, Slaby BM^1#^

# SAR324

## Central metabolism

The near-complete gylcolysis pathway leading to pyruvate (Pyr), the reactions of the tricarboxylic acid (TCA) cycle and some conversions of the pentose phosphate pathway (PPP) were annotated. The enzyme phosphoenolpyruvate (PEP) carboxykinase was present, that is usually involved in gluconeogenesis, but has been shown to act in catabolic CO_2_ fixation in the obligate anaerobe *Anaerobiospirillum succiniciproducens* (1). It is unclear if CO_2_ fixation using this way occurs in *V. pourtalesii*-associated SAR324, but a similar pathway was proposed for the crenarchaeal species *Ignicoccus hospitalis* where carbon is fixed via succinyl-CoA in a partial reductive citric acid cycle, which might be coupled with the 4-hydroxybutyrate route of acetyl-CoA regeneration (2). However, no genes involved in the 4-hydroxybutyrate cycle or other carbon fixation pathways such as the Wood-Ljungdahl pathway were found here. Thus, the resulting Oxalate (Oxa) may fuel the TCA cycle instead. Pyruvate (Pyr) is usually converted aerobically into acetyl-CoA by the pyruvate dehydrogenase enzyme complex, which is encoded in the SAR324 MAGs.

SAR324 genomes encode the glyoxylate-bypass of the TCA cycle (Error: Reference source not found5A: orange arrows within the TCA cycle), which is required by bacteria to grow anaerobically on fatty acids and acetate (3). A potential AMP-dependent acetyl-CoA synthetase (IPR000873) to utilize acetate was identified in these genomes as well, whereas not all enzymes required for the utilization of fatty acids were detected: for example, acyl-CoA oxidase/dehydrogenase (*acd*) and enoyl-CoA hydratase (*HADHA*) were found, whereas long-chain enoyl coenzyme A hydratase, 3-hydroxyacyl-CoA dehydrogenase (*HADH*), acetyl-CoA acyltransferase (*FadA*), and acetyl-CoA C-acetyltransferase (*atoB*) were not detected. A number of genes involved in fatty acid biosynthesis were detected, such as acetyl-CoA, acetyl-CoA carboxylase, *ACACA*, malonyl CoA-acyl carrier protein transacylase (*fabD*), beta-ketoacyl-acyl-carrier-protein synthase II (*FabF*) and beta-hydroxyacyl-(acyl-carrier-protein) dehydratase (*FabA/Z*). Others are missing: [3-oxoacyl-[acyl-carrier-protein] synthase III (*FabH*), 3-oxoacyl-[acyl-carrier-protein] synthase I (*FabB*), acetoacetyl-[acyl-carrier protein] synthase (*FabY*), 3-oxoacyl-[acyl-carrier protein] reductase (*FabG*), enoyl-[acyl-carrier protein] reductase I (*FabI*), enoyl-[acyl-carrier protein] reductase II (*FabK*)]. Therefore, it is not clear if SAR324 bacteria are able to synthesize and/ or utilize fatty acids. Genes involved in assimilatory sulfate reduction were detected in SAR324 MAGs but the pathway was not annotated completely. A facultative anaerobic lifestyle is supported by the presence of lactate dehydrogenase (LHD) involved in fermentation (3).

## Respiration

SAR324 could gain energy by aerobic and anaerobic respiration: the MAGs encoded subunits of a NADH:ubiquinone oxidoreductase (complex I), fumarate reductase/succinate dehydrogenase (complex II), and cytochrome c oxidase-like (complex IV) subunits of a F0F1-(f-type) ATPase (complex V). Subunits of complex III were not detected and it is unclear if the respiratory chain can be functional without this enzyme complex or if it was missed during annotation. Gene homologs of succinate dehydrogenase/ fumarate reductase are referring to succinate:quinone reductase (SQR, or complex II) and quinol:fumarate reductase (QFR) collectively (4). While SQR is found in aerobic organisms (catalyzing the oxidation of succinate to fumarate in the citric acid cycle and donating the electrons to quinone in the membrane), QFR can be found in anaerobic cells respiring with fumarate as terminal electron acceptor. SQR and QFR are very similar in composition and structure, but catalyze opposite reactions *in vivo*. They may have evolved from a common ancestor, and in *E. coli* they are capable of functionally replacing each other (5). Thus, both aerobic respiration and anaerobic fumarate respiration may be functional in SAR324. We found several nitrite/ sulfite reductase genes, which confirms their capability of (at least facultative) anaerobic respiration using sulfite (or nitrite) as electron acceptor in addition to the above-mentioned fumarate respiration. A p-type ATPase was annotated that may be driven by cations for additional energy generation or that may utilize ATP for the export of cations.

## Amino acid biosynthesis

Above described central metabolic pathways glycolysis, TCA cycle and PPP provide energy and important precursors for amino acid, vitamin (vit) and co-factor biosynthesis. Phenylalanine (Phe), tyrosine (Tyr) and tryptophan (Trp) are synthesized from erythrose-4-phosphate (E4P) and phosphoenol pyruvate (PEP) in SAR324. Additionally, they may be synthesized from fructose-1,6-bisphosphate (F1,6P_2_). 5-phosphoribosyl diphosphate (PRPP) resulting from the PPP serves as an important precursor for the biosynthesis of purines and pyrimidines as well as histamine (His). Pyruvate (Pyr) is precursor for the biosynthesis of valine (Val), isoleucine (Ile), leucine (Leu). The enzyme alanine dehydrogenase to synthesize alanine (Ala) was not annotated. Intermediates of the TCA cycle are also precursors for amino acid synthesis: 2-Oxoglutarate (2Og) serves as substrate for the assimilation of inorganic nitrogen and the synthesis of glutamic acid (Glu), arginine (Arg), and glutamine (Gln). Aspartic acid (Asp) can be synthesized from fumarate (Fur), and threonine (Thr) from Asp.

## Biosynthesis of b-vitamins

SAR324 synthesize different B vitamins and co-factors: thiamine pyrophosphate (TPP), an essential co-factor of central metabolism and amino acid biosynthesis, is derived from thiamine (vitB_1_). The biosynthesis of thiamin could not be fully resolved in SAR324, but the essential TPP could be produced with pyridoxal as substrate using SsuA/THI5-like enzyme, phosphomethylpyrimidine kinase and thiamine phosphate synthase. The biosynthesis of riboflavin (vitB_2_) from purines and Rl5P is partially encoded; riboflavin serves as precursor for the biosynthesis of the coenzyme F420, which is possibly involved in redox reactions. The biosynthesis of folate (vitB_9_) from GTP is partially encoded in SAR324. Via the enzyme dihydrofolate reductase, folate is converted into tetrahydrofolate (THF) representing a one-carbon unit carrier necessary in a variety of biosynthetic reactions. Pantotheonate (vitB_5_) can likely be synthesized from Pyr. VitB_5_ is required by animals in order to synthesize coenzyme-A (CoA) and to synthesize and metabolize proteins, carbohydrates, and fatty acids. The sponge host could, thus, benefit from the vitamin production of its SAR324 symbionts.

## Transporters

*V. pourtalesii*-associated SAR324 encode a large variety of transporters of different classes. The Tat and Sec protein excretion systems were annotated, the most highly conserved mechanisms of protein secretion to the periplasm or inner membrane (reviewed in (6)). As the Tat pathway has been shown to play a role in pathogenic (for full virulence in animal infection models) but also non-pathogenic symbionts, we hypothesize that it may play a role in the interaction between microbes and sponge host as well. The Sec and Sec(B) pathways together secrete proteins into the periplasm or out of the cell, while proteins meant to remain in the inner membrane are transported via the SRP pathway using the FtsY docking protein and the SecYEG channel (6). In SAR324 we detected the integral membrane complex (*SecY*, *SecE* and *SecG*), and two additional genes encoding membrane proteins that promote the release of the mature peptide into the periplasm (*SecD* and *SecF*). *YidC* and *YajC* play a part in preprotein translocase activity. However, the chaperone (*SecB*) was not detected. SAR324 are able to excrete effector proteins with *SecA* serving as ATPase. Additionally, in SAR324 *FtsY* and *ffh* (prokaryotic counterpart of SRP) are found, translocating unfolded proteins in the membrane. Proteins transported by Sec and Tat pathways may subsequently be transported out of the cell with the help of another secretion system (6).

We found a *ShlB*-like protein typical for type V secretion systems in SAR324. Additionally, they contain evidence for type I and II secretion systems (T1SS, T2SS), typically found in gram negative bacteria. Such secretion systems are known to transport small molecules like antibiotics and toxins (T1SS) and folded proteins from the periplasm into the extracellular environment (T2SS). Proteins secreted via a T2SS channel through the outer membrane must first be delivered to the periplasm via the Sec or Tat secretion pathways (6).

Transporters belonging to the so-called Major Facilitator Superfamily (MFS) were annotated. MFS is a large and diverse protein superfamily that includes millions of sequenced members, and can catalyze uniport, solute:cation (H+, but seldom Na+) symport and/or solute:H+ or solute:solute antiport. They may be involved in multi drug efflux and carbohydrate transport in SAR324 (*AraJ, EmrB, QacA*). Further ‘drug efflux’-related transporters (DMT-class: *EamA, RhaT, EmrE, RarD*; ABC-class: *MdlB*-like; MOP- and RND-classes) were found.

We detected transporters likely involved in osmoregulation, such as *GlpF*-like MIP transporter, *BetT*-like BCCT transporter and *ProXVW*-like ABC transporter to import osmoregulatory substances as well as a transporter of the MscS-class for the export of relevant ions and/ or substances. SAR324 cells might reduce the effects of toxic metals using Fluc- (*CrcB*) and HCC-like transporter (*HlyC*) by exporting Co^2+^ and F^-^ ions.

Diverse transporter classes were detected that are likely involved in import or exchange of ions: NhaC for Na^+^:H^+^ exchange; PNaS for K^+^:Na^+^ symport; CaCA for Ca^2+^:Na^+^ antiport; CIC (*EriC*) for Cl^-^ import; TrK, VIC and YggT for K^+^ import; Amt (*AmtB*) for NH_4_^+^ import in one MAG; ABC-transporter (*PstAB*) for PO_4_^3-^ and phosphonate import; ABC transporter (*ZnuB*- and *TroCD*-like) for Mg^2+^, Mn^2+^ and/ or Zn^2+^ import. Additionally, genes for substrate-binding proteins as part of an ABC transporter were annotated, which might be involved in SO_4_^2-^, NO_3_^-^ or urea uptake.

Several transporters were annotated for amino acids uptake (ABC: *His*-like, *ProW*-like, *Art*-like, *LivFGMHKBP*) and efflux (LysE/ RhtB and LivE: *AzlC*), peptide (ABC: *Opp/Dpp*-like), and carbohydrates transport. A TRAP-transporter (*DctMQP*) was identified in SAR324 MAGs to take up C4-dicarboxylates (fumarate, malate, succinate), which could feed the TCA cycle under aerobic conditions, and fumarate respiration under anaerobic conditions (7). A tricarboxylate (TTT: *TctABC*) transporter was annotated in the most complete SAR324 MAG (SAR324_126). TTT-transporters are known for ion-dependent citrate import, possibly feeding the TCA cycle (8). In one MAG, a transporter of the SSPTS class (*PstN*) was identified.

We detected a *ComEC*-like transporter (DNA-T class) which is known to be involved in DNA uptake. In the symbiosis context, it is interesting to note that SAR324 MAGs contained AI-2E family transporters, a large family of prokaryotic proteins derived from a variety of bacteria and archaea, sometimes in multiple paralogues per genome. There is strong evidence, that Al-2E family homologues function as an autoinducer-2 (AI-2) exporter in *E. coli* cells to control biofilm formation. AI-2 is a proposed signaling molecule for interspecies communication in bacteria (reviewed in (9)) and we detected copies of the *luxS* gene, the proposed AI-2 synthetase, in all three SAR324 genomes (9).

## Cell wall/ membrane/ motility/ chemotaxis

Genes involved in phospholipid-, peptidoglycan- and lipopolysaccharide biosynthesis as well as the LPT exporter were identified in SAR324 MAGs, typical properties of gram-negative bacterial membranes and cell walls. We detected a *LolABDCDE* transporter system which, e.g., in *E. coli* catalyzes the localization of lipoproteins (lipid A) to the outer membrane (10), and the *MlaFEDB* complex which is likely involved in maintaining the asymmetrical gram-negative outer membrane via retrograde phospholipid transport (11). Enzymes involved in lipid A assembly (*LpxA, LxpB, LpxC, LxpH, LxpB, LxpK*) were detected in all SAR324. LPS is exported by inner-membrane *LptBFGC* ABC-transporters. The outer membrane translocation unit *LptD/E* and the periplasmatic acting *LptA* were, however, not annotated. SAR324 showed all signatures of a typical gram-negative cell. In genomes of free-living SAR324 signatures for flagellum formation and chemotaxis have been found previously (12). Both properties are missing in *V. pourtalesii*-associated SAR324, which is in line with previous metagenomic and MAG studies of other sponge symbionts (e.g., (13), and could be an adaption to life within the animal host.

# Crenarchaeota

## Central metabolism

The pathways of the central metabolism of the four crenarchaeal MAGs were very similar to those of the above-described SAR324. The complete glycolysis pathway resulting in pyruvate (Pyr) was annotated, as well as the reactions of the tricarboxylate acid (TCA) cycle, except the gene(s) encoding for the conversion of 2-Oxogluterate (2Ox) into Succinyl-CoA (SCoA). Acetyl-CoA (AcoA) is produced anaerobically using a pyruvate ferredoxin/flavodoxin oxidoreductase which catalyzes the oxidative decarboxylation of pyruvate with the participation of thiamine, then an acetyl moiety is transferred to CoA for the synthesis of acetyl-CoA (14). Some conversions of the pentose phosphate pathway (PPP) were identified: the enzyme PEP (phosphoenolpyruvate) carboxykinase was present, that is usually involved in gluconeogenesis. As described for SAR324, this enzyme could be involved in CO_2_-fixation (1, 2). Also similar to SAR324, there is no evidence for autotrophic carbon fixation pathways such as the Wood-Ljungdahl pathway, the 3-hydroxypropionate bicycle or the hydroxypropionate-hydroxybutyrate cycle.

The enzymes enoyl-CoA hydratase (*HADHA*), 3-hydroxyacyl-CoA dehydrogenase (*HADH, FadJ*, *FadB*) involved in fatty acid degradation were annotated, but long-chain enoyl coenzyme A hydratase, acetyl-CoA acyltransferase (*FadA*), and acetyl-CoA C-acetyltransferase (*atoB*) were not detected. Genes involved in fatty acid biosynthesis from acetyl-CoA are missing. Therefore, it is not clear if the crenarchaeal symbionts are able to utilize fatty acids, but fatty acid biosynthesis is implausible.

The complete gene repertoire for assimilatory sulphate reduction (Figure 5B) into the amino acids cysteine (Cys) and serine (Ser) was annotated. A facultatively anaerobic lifestyle is supported by the presence of lactate dehydrogenase (LHD) involved in fermentation as described for SAR324 MAGs (3).

## Respiration

Crenarchaeota gain energy by aerobic and possibly also anaerobic respiration. Subunits of a NADH:ubiquinone oxidoreductase (complex I), fumarate reductase/succinate dehydrogenase (complex II), and cytochrome c oxidase-like (complex IV) subunits of a F0F1-(f-type) ATPase (complex V) were encoded in the genomes. Subunits of complex III were not detected. Gene homologs of succinate dehydrogenase/ fumarate reductase are referring to succinate:quinone reductase (SQR, or complex II) and quinol:fumarate reductase (QFR) collectively (4). These are possibly active under aerobic conditions (catalyzing the oxidation of succinate to fumarate in the citric acid cycle and donating the electrons to quinone in the membrane), or in anaerobic phases (involved in respiration with fumarate as terminal electron acceptor). We found several nitrite/ sulfite reductase genes, which confirms that in Crenarchaeota – similar to SAR324 - at least facultatively anaerobic respiration using sulfite (or nitrite) as electron acceptor may be functional in addition to fumarate respiration. In the Crenarchaeota a (possibly K^+^-independent) H^+^-pyrophosphatases (PPase) was annotated, which could hydrolyze one pyrophosphate per translocated H^+^ and make use of H^+^ brought into the cell by ATPase (complex V) of the respiratory chain. At the same time, it would provide sufficient amounts of phosphate (Pi) for the ATP synthesis and therefore be a part of an energy-conserving system (15).

The AMO Operon (*AmoABC*) was annotated in the genomes, suggesting that they are capable of aerobic ammonia oxidation, which has been published for other marine and sponge-associated Crenarchaeota.

## Amino acid biosynthesis

Phenylalanine (Phe), tyrosine (Tyr), and tryptophan (Trp) are synthesized from erythrose-4-phosphate (E4P) and phosphoenol pyruvate (PEP) in the Crenarchaeota MAGs. Additionally, they may also be synthesized from fructose-1,6-bisphosphate (F1,6P_2_). The 5-phosphoribosyl diphosphate (PRPP) resulting from the PPP serves as an important precursor for the biosynthesis of purines and pyrimidines as well as histamine (His). Pyruvate (Pyr) is precursor for the biosynthesis of valine (Val), isoleucine (Ile), leucine (Leu) and alanine (Ala). Intermediates of the TCA cycle are also precursors for amino acid synthesis: 2-Oxoglutarate (2Og) serves as substrate for assimilation of inorganic nitrogen and the synthesis of glutamic acid (Glu), arginine (Arg), glutamine (Gln), and proline (Pro) is encoded. Aspartic acid (Asp) can be synthesized from fumarate (Fur), and threonine (Thr) and lysine (Lys) from Asp. Cystein (Cys) and serine (Ser) are products of assimilatory sulfate reduction. The amino acids tryptophan (Trp) and glycine (Gly) can be synthesized from Ser. Crenarchaeota seem to be able to synthesize fewer amino acids than SAR324.

## Biosynthesis of b-vitamins

Thiamine pyrophosphate (TPP), an essential co-factor of central metabolism and amino acid biosynthesis, is derived from thiamine (vitB_1_). The biosynthesis of from aminoimidazole ribotide (AIR; purine metabolism) thiamin is almost completely encoded in the crenarchaeal genomes, but not from the vitamin pyridoxal like in SAR324. The biosynthesis of vitamin B_6_ (Pyridoxal derivates) from glyceraldehyde-3-phosphate (Ga3P) and ribulose-5-phosphate (Rl5P) is encoded. The biosynthesis of riboflavin (vitB_2_) from purines and Rl5P was annotated and parts of the subsequent synthesis pathway of coenzyme F420 (possibly involved in redox reactions) were present. The biosynthesis of folate (vitB_9_) from GTP is partially encoded. Since the enzyme dihydrofolate reductase was not found, the conversion of folate into THF representing a one-carbon unit carrier necessary in a variety of biosynthetic reactions, could not be fully resolved. Pantotheonate (vitB_5_) can likely be synthesized from Pyr, but the pathway remains incomplete. As described for SAR324, VitB_5_ could be provided to the animal host and/or to other members of the microbial symbiotic community. The synthesis of nicotinate (vitB_3_) from aspartate (Asp) is fully encoded. Cobalamine coenzyme (vitB_12_) can be synthesized via an aerobic or an anaerobic pathway from the same precursor dihydrosirohydrochlorin (16). Interestingly, only genes involved in the anaerobic pathway were found in crenarchaeal MAGs. The pathway was, however, not completely encoded. Additionally, vitB_12_ might be synthesized using the partially present energy efficient salvage pathway from cobinamide which can be imported by an ABC transporter (*BtuCDF*) (16).

## Transporters

A number of different transporter classes were detected, such as the *Tat* and *Sec* protein excretion systems. Using the *Sec* and *Sec(B)* pathways, Crenarchaeota may be able to secrete unfolded proteins which might be translocated outside or into the periplasmatic space (*YajC*)*.* Some genes were, however, not detected (*YidC*, *SecB, SecA*). The presence of genes *SRP* and *FtsY* indicates that some proteins might remain in the membrane. In both options the SecYEG channel and the promoting membrane proteins *SecD* and *SecF* (not detected) might be used (see description in SAR324 part). As no outer membrane pore was detected, it remains unclear, whether effector molecules are translocated to the extracellular environment. In contrast to SAR324, no other protein secretion systems (T1SS, T2SS or T5SS) were annotated in crenarchaeal MAGs.

We detected two Major Facilitator Superfamily (MFS) transporters (*EmrB/ QacA*), which are probably involved in the transport of small solutes, glycerol-3-phosphate, or in multidrug efflux. Additionally, a transporter of the class DMT (*RhaT*) is possibly used for the import of metabolites, which could not be identified further. Transporters involved in osmoregulation are encoded, such as a MscC-class exporter for relevant molecules and ions, and an ABC transporter for the import of glycine and betaine (not completely annotated).

Diverse transporter classes were detected, which are likely involved in import or exchange of ions: ABC transporters (*ZnuB*- and *TroCD*-like) for Mg^2+^, Mn^2+^ and/ or Zn^2+^ import; ABC-transporter (*PstABCS*) for PO_4_^3-^ and phosphonate import; ZIP transporter for Zn^2+^, Fe^2+^ or Mn^2+^ import; a CaCa antiporter (Ca^2+^: Na^+^); TrK for K^+^ import; Nramp (*MntH*) for Mn^2+^ or Fe^2+^ import; FeoB for Fe^2+^ import; MIT transporter (*CorA, HlyC*) for Mg^2+^, Co^2+^ or Ni^2+^ exchange; CPA2 transporter (*NhaP, KefB*) for H^+^:K^+^ (or Na^+^) antiport; SBT transporter for carbon of Na^+^ exchange; TRIC transporter for anion exchange; and Amt (*AmtB*) for NH_4_^+^ import. Additionally, we detected genes for substrate-binding proteins as part of an ABC transporter, which might be involved in SO_4_^2-^, NO_3_^-^ and taurine uptake. Some transporters for amino acids transport were annotated (ABC: *LivFGMH* for leucine, isoleucine and valine import; DAACS for glutamate or aspartate: H^+^ or Na^+^ symport; RhtB for homoserine, threonine antiport) and for peptides (ABC: *Opp/Dpp*-like). Like in SAR324 MAGs, an importer for dicarboxylates (DASS class) was identified in Crenarchaeota, which supports the hypothesis that sustaining the TCA cycle or importing substrates for fumarate respiration may be crucial in this environment (7). Additionally, subunits of diverse other ABC transporters were annotated (for the transport of e.g. lipoprotein polysaccharides, and polyamide) which were not complete and the functionality remains unclear.

## Cell wall/ membrane/ motility/ chemotaxis

In the crenarchaeal MAGs, no genes involved in membrane biosynthesis, motility or chemotaxis were annotated.

# References

1. Podkovyrov SM, Zeikus JG. 1993. Purification and characterization of phosphoenolpyruvate carboxykinase, a catabolic CO_2_-fixing enzyme, from *Anaerobiospirillum succiniciproducens*. J Gen Microbiol 139:223–228.

2. Podar M, Anderson I, Makarova KS, Elkins JG, Ivanova N, Wall MA, Lykidis A, Mavromatis K, Sun H, Hudson ME, Chen W, Deciu C, Hutchison D, Eads JR, Anderson A, Fernandes F, Szeto E, Lapidus A, Kyrpides NC, Saier MH, Richardson PM, Rachel R, Huber H, Eisen JA, Koonin EV, Keller M, Stetter KO. 2008. A genomic analysis of the archaeal system *Ignicoccus hospitalis*-*Nanoarchaeum equitans*. Genome Biol 9:R158.

3. White D, Drummond J, Fuqua C. 2012. The physiology and biochemistry of prokaryotes. Oxford University Press.

4. Hägerhäll C. 1997. Succinate: Quinone Oxidoreductases. Variations on a conserved theme. Biochim Biophys Acta 1320:107–141.

5. Maklashina E, Berthold DA, Cecchini G. 1998. Anaerobic expression of *Escherichia coli* succinate dehydrogenase: functional replacement of fumarate reductase in the respiratory chain during anaerobic growth. J Bacteriol 180:5989–5996.

6. Green ER, Mecsas J. 2016. Bacterial secretion systems: an overview. Microbiol Spectr 4:VMBF-0012-2015.

7. Unden G, Strecker A, Kleefeld A, Kim O Bin. 2016. C4-dicarboxylate utilization in aerobic and anaerobic growth. Ecosal Plus 7:1-33.

8. Rosa LT, Bianconi ME, Thomas GH, Kelly DJ. 2018. Tripartite ATP-independent periplasmic (TRAP) transporters and tripartite tricarboxylate transporters (TTT): from uptake to pathogenicity. Front Cell Infect Microbiol 8:33.

9. Miller MB, Bassler BL. 2001. Quorum sensing in bacteria. Annu Rev Microbiol 55:165–199.

10. Okuda S, Tokuda H. 2011. Lipoprotein sorting in bacteria. Annu Rev Microbiol 65:239–259.

11. Hughes GW, Hall SCL, Laxton CS, Sridhar P, Mahadi AH, Hatton C, Piggot TJ, Wotherspoon PJ, Leney AC, Ward DG, Jamshad M, Spana V, Cadby IT, Harding C, Isom GL, Bryant JA, Parr RJ, Yakub Y, Jeeves M, Huber D, Henderson IR, Clifton LA, Lovering AL, Knowles TJ. 2019. Evidence for phospholipid export from the bacterial inner membrane by the Mla ABC transport system. Nat Microbiol 4:1692–1705.

12. Chitsaz H, Yee-Greenbaum JL, Tesler G, Lombardo M-J, Dupont CL, Badger JH, Novotny M, Rusch DB, Fraser LJ, Gormley NA, Schulz-Trieglaff O, Smith GP, Evers DJ, Pevzner PA, Lasken RS. 2011. Efficient de novo assembly of single-cell bacterial genomes from short-read data sets. Nat Biotechnol 29:915–921.

13. Pita L, Rix L, Slaby BM, Franke A, Hentschel U. 2018. The sponge holobiont in a changing ocean: from microbes to ecosystems. Microbiome 6:46.

14. Kletzin A, Adams MWW. 1996. Molecular and phylogenetic characterization of pyruvate and 2-ketoisovalerate ferredoxin oxidoreductases from *Pyrococcus furiosus* and pyruvate ferredoxin oxidoreductase from *Thermotoga maritima*. J Bacteriol 178:248–257.

15. Bäumer S, Lentes S, Gottschalk G, Deppenmeier U. 2002. Identification and analysis of proton-translocating pyrophosphatases in the methanogenic archaeon *Methanosarcina mazei*. Archaea 1:1–7.

16. Fang H, Kang J, Zhang D. 2017. Microbial production of vitamin B12: a review and future perspectives. Microb Cell Fact 16:15.
